# Supplementary material for: Application of Curcumin Emulsion Carrier from Ultrasonic-Assisted Prepared Octenyl Succinic Anhydride Rice Starch
Source: Molecules. 2022 Oct 17;27(20):6955. doi: 10.3390/molecules27206955 (PMC9612171; doi:10.3390/molecules27206955)
Supplement: Supplementary file 1 [file molecules-27-06955-s001.zip › molecules-1913230-supplementary.pptx]

## Slide 1
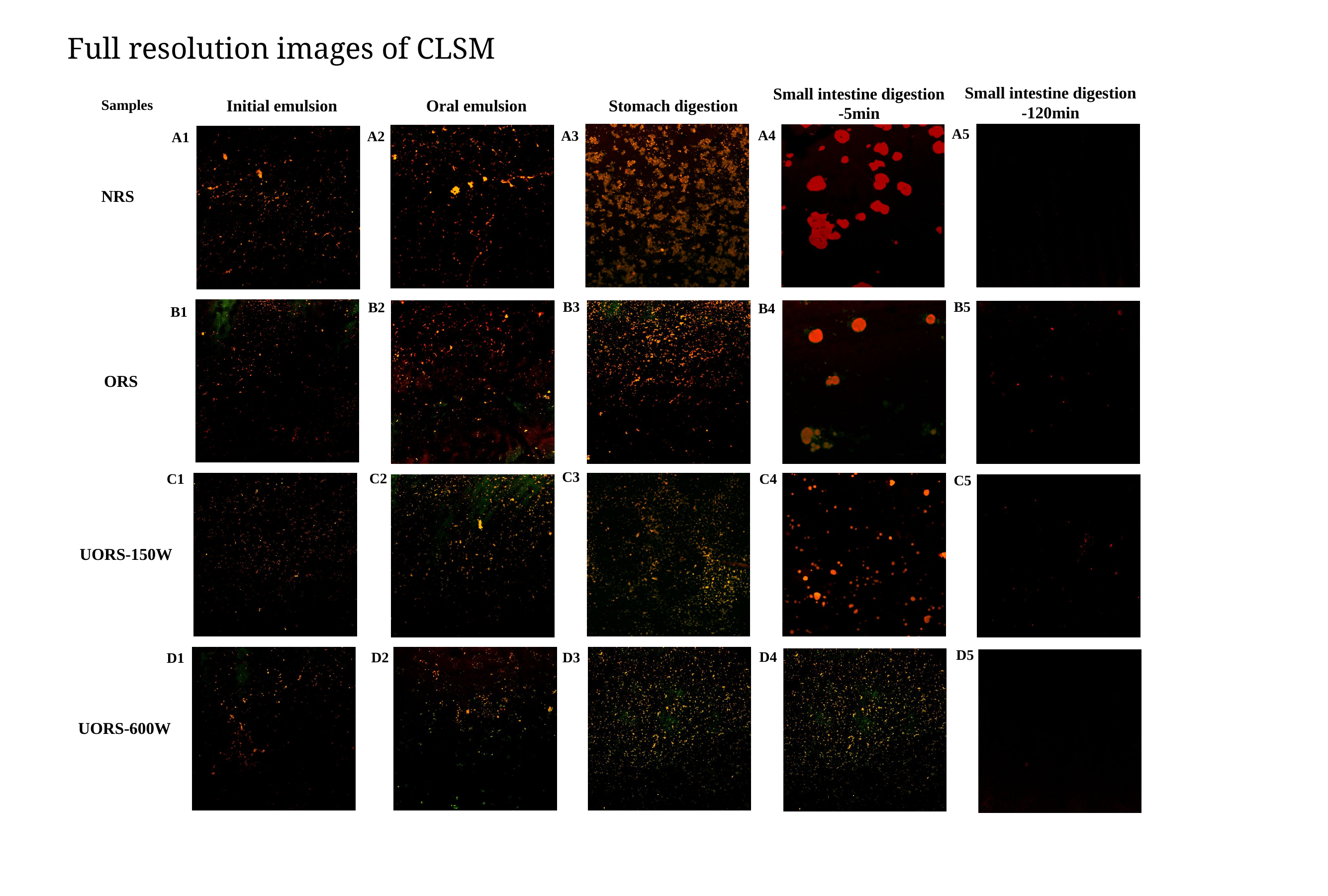

Full resolution images of CLSM
Small intestine digestion
-120min
Small intestine digestion
-5min
Initial emulsion
Stomach digestion
Oral emulsion
Samples
A5
A4
A3
A2
A1
NRS
B3
B5
B2
B4
B1
ORS
C3
C2
C1
C4
C5
UORS-150W
D5
D4
D2
D3
D1
UORS-600W
